# Two Proposals of a Simple Analog Conditioning Circuit for Remote Resistive Sensors with a Three-Wire Connection

**DOI:** 10.3390/s24020422

**Published:** 2024-01-10

**Authors:** Ferran Reverter

**Affiliations:** Department of Electronic Engineering, Universitat Politècnica de Catalunya—BarcelonaTech, 08860 Castelldefels, Spain; ferran.reverter@upc.edu; Tel.: +34-934-137-076

**Keywords:** parasitic resistance, remote sensor, resistive sensor, sensor interface electronics, thermal sensor

## Abstract

This article proposes and experimentally characterizes two implementations of a novel front-end circuit for three-wire connected resistive sensors with a wire-resistance compensation. The first implementation relies on two twin diodes, whereas the second on a switch; in both cases, those devices are non-remote (i.e., they are placed at the circuit end). The two circuit proposals have a square-wave input excitation so that a constant current with the two polarities is alternatively generated. Then, depending on that polarity, the current goes through either the sensor and the wire parasitic resistances or just the parasitic resistances. This generates a square-wave bipolar output signal whose average value, which is obtained by a low-pass filter, is proportional to the sensor resistance and only depends on the mismatch between two of the three wire resistances involved. Experimental tests applied to resistances related to a Pt100 thermal sensor show a remarkable linearity. For example, the switch-based front-end circuit offers a non-linearity error lower than 0.01% full-scale span, and this is practically insensitive to both the presence and the mismatch between the wire resistances.

## 1. Introduction

Resistive sensors convert information of interest from a given energy domain (such as thermal, mechanical, chemical, optical, and magnetic domains) to the electrical domain by changing its electrical resistance. The electrical resistance of the sensor changes because the measurand alters the resistivity, length, and/or cross-section area of the sensor material. Resistive sensors are very commonly employed in electronic measurement systems to monitor temperature (e.g., platinum sensors such as the typical Pt100 that offers 100 Ω at 0 °C), light (e.g., light-dependent resistors, LDR), gas concentration (e.g., tin dioxide gas sensors), mechanical stress (e.g., strain gauges), and humidity [1]. Also, resistive sensors have been proposed to measure humans’ heart rate [2].

In industry applications, we can have scenarios in which the sensor is located at a certain distance (up to several meters) from the read-out electronic circuit; these are known as remote sensors. This situation occurs when the sensing region offers a harsh environment with extreme operating conditions. A typical scenario is when the sensor is located in an environment with very low or very high temperatures that are not withstood by standard silicon chips (such as operational amplifiers, OpAmp) required in the read-out circuit. For example, a Pt100-type thermal resistive sensor measuring temperatures below −50 °C and/or above +150 °C has to be placed remotely since the operating temperature range of the commercial chips is, in the best case, between −50 °C and +150 °C; this is a typical example where the circuits proposed herein could be applied. Another scenario is when the sensor has to be located in an environment with high radiation levels [3].

Many electronic interface circuits for resistive sensors can be found in the literature. According to [4], these circuits can be classified in five groups: (1) voltage divider [5] and bridge circuits (e.g., Wheatstone bridge); (2) circuits based on a current source (e.g., Howland current source); (3) resistance-to-frequency converters (e.g., 555 timer-based oscillator); (4) resistance-to-phase converters; and (5) direct interface circuits (DIC), which do not have any active device between the sensor and the digital processing unit [6]. For the digitization of the information, circuits that belong to groups 1 and 2 require an analog-to-digital converter, whereas those that belong to groups 3, 4, and 5 usually employ a digital timer that performs a time-to-digital conversion. The concepts associated to groups 1 and 5 have been recently mixed in [7], thus resulting in a DIC that measures a voltage divider with three output terminals. However, most of the previous circuits are exclusively valid for non-remote resistive sensors.

For the measurement of remote resistive sensors, other circuit topologies with a wire-resistance compensation have been proposed in the last years [8,9,10,11,12,13,14,15,16,17]. The main objective of these circuits is to provide an output signal insensitive to the temperature-dependent parasitic resistance of the interconnecting wires. Note that, without an appropriate wire-resistance compensation, the effects of the parasitic resistances can be quite critical, especially when the sensor has a low resistance (e.g., a Pt100 thermal sensor) and the interconnecting cable is long (with a typical parasitic resistance of 0.3–0.4 Ω/m [8]). In that sense, different wire-resistance compensation techniques applied to resistive sensors with a two-wire [8,9,10,11], three-wire [12,13,14,15], and four-wire [14,16,17] interconnection can be found in the recent literature.

As for circuits intended for three-wire connected resistive sensors, which is the focus of this paper, three types of front-end circuits can be distinguished: (i) Analog circuits, which are designed with analog active devices, such as an OpAmp, and provide an analog output signal. For example, a circuit based on two OpAmps with a maximum non-linearity error (NLE) of 0.24% when measuring a Pt100 was suggested in [14]. Its output signal depended on the mismatch between two wire resistances (wires 1–3 using the terminology in [14]) provided that two resistors of the circuit were well matched. (ii) Mixed circuits, in which the core is a mixed analog/digital device, such as a comparator, to build a relaxation oscillator with a time-based output signal. For instance, the relaxation oscillator in [12] was based on a comparator, an OpAmp, two switches, and a programmable-gain amplifier (PGA), whereas that in [13], on a comparator and four switches. In [12], the output signal was only affected by the mismatch between two of the three wire resistances involved, whereas in [13], by the mismatch between two couples of wire resistances (to be precise, wires 1–2 and 2–3, using the terminology employed in [13]). The experimental test of both circuits [12,13] showed a maximum NLE of 0.09%. And (iii) digital circuits, in which the measurement is directly carried out by a digital active device such as a microcontroller unit (MCU). For example, the circuit proposed in [15] had a maximum NLE of around 0.03% full-scale span (FSS). Further, the output depended only on the mismatch between two wire resistances (wires 1–2 according to the terminology used in [15]).

In this paper, two implementations of a novel, simple front-end circuit for three-wire connected resistive sensors are proposed. The two circuits belong to the analog group, thus providing an analog signal at the output, as in [14]. In addition, the sensor is excited by a constant current, which ensures a linear output signal [18] and also enables a better control of the self-heating effects [14]. In the first proposed implementation, the wire-resistance compensation is carried out by using a couple of twin diodes, as in [8,11]. However, unlike [8,11], here, the two diodes are not remote but placed within the circuit. In the second circuit implementation, the diodes are replaced by a switch controlled by the input signal.

The organization of the paper is as follows. The operating principle of the two proposed implementations of the circuit is explained in Section 2. The materials and method applied to test the circuits are reported in Section 3. The experimental results and their discussion are provided in Section 4. To conclude, the main conclusions are given in Section 5.

## 2. Operating Principle

This section explains the operating principle of the two circuits proposed for remote resistive sensors (*R*_x_) with a three-wire connection, whose parasitic resistances are modelled by *R*_w1,_ *R*_w2_, and *R*_w3_. Both circuit implementations rely on an inverting-amplifier topology with a square-wave excitation applied to the input, and a low-pass filter (LPF) placed at the output, as in [11]. The input signal has an amplitude of ±*V*_ref_, a period of *T*, and a duty cycle of 50%.

### 2.1. Diode-Based Circuit

**Figure 1:**
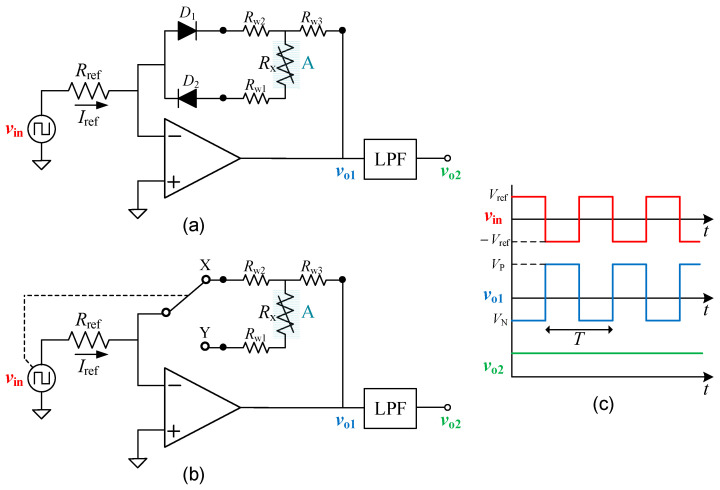
Proposed front-end circuit for a three-wire connected resistive sensor. (**a**) First proposal based on a couple of twin diodes. (**b**) Second proposal based on a switch. (**c**) Waveform of the voltage at the main nodes in the circuits shown in (**a**,**b**).

Figure 1a shows the first circuit proposed for three-wire connected resistive sensors. The circuit relies on two diodes (*D*_1_ and *D*_2_) in the feedback path of the inverting amplifier topology, as in [11]. However, unlike [11], the diodes are not remote here; only the sensor is remote, as represented in Figure 1a through the operating region “A”. Thanks to that, the remote placement of the diodes is no longer a problem [8,11]. Further, it is easier to achieve the same operating temperature in both diodes and hence a better matching in their forward voltage (*V*_F1_ and *V*_F2_, respectively). In addition, the three-wire connection of the sensor facilitates a specific interconnection to the circuit (i.e., wire #1 connected to *D*_2_ anode, wire #2 connected to *D*_1_ cathode, and wire #3 connected to OpAmp output) that enables a simple but smart wire-resistance compensation.

The operating principle of the circuit in Figure 1a is explained next by distinguishing what occurs in the positive semicycle from what happens in the negative semicycle of the bipolar input signals (*v*_in_). In the positive phase, an input current equal to *I*_ref_ = *V*_ref_/*R*_ref_ is generated, where *R*_ref_ is a reference resistor. This current then goes towards the OpAmp output through *D*_1_, *R*_w2_, and *R*_w3_. This causes a negative voltage at the OpAmp output that can be expressed as

(1) $$V_{N}=-\left[\frac{V_{\mathrm{ref}}}{R_{\mathrm{ref}}}\left(R_{w2}+R_{w3}\right)+V_{F1}\right].$$

In the negative phase, the input current has the same value but opposite direction (i.e., *I*_ref_ = −*V*_ref_/*R*_ref_), and this comes from the OpAmp output circulating via *R*_w3_, *R*_x_, *R*_w1,_ and *D*_2_. This causes a positive voltage at the OpAmp output equal to

(2) $$V_{P}=\frac{V_{\mathrm{ref}}}{R_{\mathrm{ref}}}\left(R_{x}+R_{w1}+R_{w3}\right)+V_{F2}.$$

According to the previous explanation, the output voltage (*v*_o1_) of the OpAmp is a square-wave bipolar signal with a positive (negative) amplitude equal to *V*_P_ (*V*_N_) and with inverted polarity with respect to the input, as depicted in Figure 1c. This signal, with a period *T* equal to that of the input signal, has the following average value:

(3) $$\bar{V_{o1}}=\frac{1}{T}\int_{0}^{T}v_{o1}(t)dt=\frac{1}{T}\left[\int_{0}^{T/2}V_{P}dt+\int_{T/2}^{T}V_{N}dt\right]=\frac{V_{P}+V_{N}}{2}.$$

Since the forward current of *D*_1_ and *D*_2_ is the same, it is reasonable in principle to assume *V*_F1_ = *V*_F2_. In such conditions, substituting (1) and (2) in (3) provides

(4) $$\bar{V_{o1}}=\frac{V_{\mathrm{ref}}}{2R_{\mathrm{ref}}}{(R}_{x}{+R}_{w1}-R_{w2}),$$

with an offset error that depends on the difference *R*_w1_ − *R*_w2_. In case *V*_F1_ ≠ *V*_F2_, (4) changes to

(5) $$\bar{V_{o1}}=\frac{V_{\mathrm{ref}}}{2R_{\mathrm{ref}}}{(R}_{x}{+R}_{w1}-R_{w2})+\frac{\Delta V_{F}}{2},$$

where Δ*V*_F_ = *V*_F2_ − *V*_F1_ is the mismatch in the forward voltage of the two diodes. Assuming Δ*V*_F_ = −0.2 mV [11], the offset error caused by Δ*V*_F_ is, from (5), equal to −0.1 mV.

### 2.2. Switch-Based Circuit

Figure 1b shows the second circuit proposed for three-wire connected resistive sensors. Its operating principle is very similar to that in Figure 1a, but with a single-pole double-throw switch (or 2:1 analog multiplexer) instead of the two twin diodes. The position of this switch is controlled by the input signal, which is represented with a dashed line in Figure 1b. When *v*_in_ is in the positive semicycle, the switch is at position X; whereas in the negative semicycle, it is at position Y. As in the circuit in Figure 1a, only the sensor is remote and located in an operating region “A”. With regard to the connection of the remote sensor, wire #1 is connected to the Y output of the switch, wire #2 to X output, and wire #3 to OpAmp output.

As in Section 2.1, the operating principle of the circuit in Figure 1b is explained as a function of the polarity of the input signal. In the positive semicycle of *v*_in_, a current *I*_ref_ (= *V*_ref_/*R*_ref_) goes towards the OpAmp output via *R*_sX_, *R*_w2_, and *R*_w3_, where *R*_sX_ is the parasitic on-resistance of the switch when it is at position X. This generates a negative voltage at the OpAmp output equal to

(6) $$V_{N}=-\frac{V_{\mathrm{ref}}}{R_{\mathrm{ref}}}\left({R_{sX}+R}_{w2}+R_{w3}\right).$$

On the other hand, in the negative semicycle of *v*_in_, the same current but with opposite direction (i.e., *I*_ref_ = −*V*_ref_/*R*_ref_) circulates through *R*_w3_, *R*_x_, *R*_w1,_ and *R*_sY_, where *R*_sY_ is the parasitic on-resistance of the switch when it is at position Y. This brings about a positive voltage at the OpAmp output equal to

(7) $$V_{P}=\frac{V_{\mathrm{ref}}}{R_{\mathrm{ref}}}\left(R_{x}+R_{w1}+R_{w3}+R_{\mathrm{sY}}\right).$$

Therefore, similar to Section 2.1, the output voltage (*v*_o1_) of the OpAmp is a square-wave bipolar signal with a period *T* and a positive (negative) amplitude equal to *V*_P_ (*V*_N_), as shown in Figure 1c. The average value of that signal can be computed again by (3). Replacing (6) and (7) in (3) and assuming *R*_sX_ = *R*_sY_ provides exactly the same result indicated before in (4). However, in case of *R*_sX_ ≠ *R*_sY_, expression (4) must be rewritten as follows:

(8) $$\bar{V_{o1}}=\frac{V_{\mathrm{ref}}}{2R_{\mathrm{ref}}}{(R}_{x}{+R}_{w1}-R_{w2}+{\Delta R}_{s}),$$

where Δ*R*_s_ = *R*_sY_ – *R*_sX_ is the mismatch of the on-resistances of the switch. Assuming Δ*R*_s_ = 0.1 Ω, *V*_ref_ = 1 V, and *R*_ref_ = 1 kΩ (which are the experimental values applied in the following sections), the offset error generated by Δ*R*_s_ is, from (8), equal to 50 µV. This is two times lower than that obtained due to Δ*V*_F_ in Section 2.1. Accordingly, the circuit in Figure 1b seems to be more accurate than that in Figure 1a.

### 2.3. General Comments

According to (4), which is applicable to both circuit implementations proposed in Figure 1a,b, the average value is proportional to *R*_x_ and independent of *R*_w3_, but it has an offset error that depends on the difference *R*_w1_ – *R*_w2_. Consequently, wires #1 and #2 should be as similar as possible, whereas the features of wire #3 are irrelevant. This is the same situation found in conventional Wheatstone bridge circuits [18], in circuits based on a current reference and an instrumentation amplifier [19], and in novel front-end circuits [12,14,15].

Due to the mismatch between *R*_w1_ and *R*_w2_, the measurement undergoes a relative error (with respect to FSS) that can be expressed as follows:

(9) $$e_{r}\;(\%)=\frac{{\;R}_{w1}-R_{w2}}{{\;R}_{x,\max}-R_{x,\min}}\times 100,$$

where *R*_x,max_ and *R*_x,min_ are the maximum and minimum sensor resistances under test, respectively.

The average value of the OpAmp output in Figure 1a,b is then proposed to be extracted by means of an LPF whose cut-off frequency is lower than the frequency of the input signal. As represented in Figure 1c, the resulting output voltage (*v*_o2_) of the filter will be a DC voltage equal to $\bar{V_{o1}}$ that increases with increasing the sensor resistance. This voltage can be then amplified and converted to digital, as in other front-end circuits for sensors.

### 2.4. Analysis of Non-Idealities

The analysis provided in Section 2.1 and Section 2.2 has considered the use of ideal components, but the circuits proposed in Figure 1a,b can be subjected to the effect of non-idealities. The main ones are discussed in the following paragraphs.

As for the OpAmp, we have the effects of the input offset voltage (*V*_IO_). The analysis of the two proposed circuits assuming *V*_IO_ results in the following approximated expression of the output voltage:

(10) $$\bar{V_{o1,V_{IO}}}\approx \frac{\left(V_{\mathrm{ref}}+V_{\mathrm{IO}}\right)}{2R_{\mathrm{ref}}}R_{x}+V_{\mathrm{IO}}$$

This has been obtained assuming Δ*V*_F_ = 0 in the diode-based circuit, Δ*R*_s_ = 0 in the switch-based circuit, and *R*_w1_ = *R*_w2_ and *R*_ref_ much higher than the wire parasitic resistances in both circuits. From (10), *V*_IO_ causes sensitivity and offset errors. Considering *V*_IO_ = ±300 µV, which is the typical value of the OpAmp used later, the offset error is ±300 µV and the sensitivity error is ±0.03%. The input bias current (*I*_IB_) of the OpAmp also generates sensitivity and offset errors, but these are much smaller (at least, a factor of 10^5^) than those caused by *V*_IO_ if an OpAmp with a low *I*_IB_ (in the pA range) is selected [11].

As for the input signal source, we have the effects of its output resistance (*R*_out_). In Section 2.1 and Section 2.2, *R*_out_ was considered to be negligible with respect to *R*_ref_. In case this assumption is not valid, the generated current would be *I*_ref_ = *V*_ref_/*R*_eq_, where *R*_eq_ = *R*_ref_ + *R*_out_, thus causing a sensitivity error. However, this limitation can be easily solved by placing an OpAmp-based voltage follower between the input source and *R*_ref_.

Several parasitic resistances (for instance, due to the tracks of the printed circuit board, the packaging of the chips, and the internal bonding of the integrated circuits) were assumed negligible in Section 2.1 and Section 2.2. Note, however, that most of these parasitic resistances are common to the two phases of the operating principle of the proposed circuits. In other words, if these parasitic resistances were considered, both *V*_P_ in (7) and *V*_N_ in (6) would have an additional resistive component with the same value. Therefore, when the average value was extracted by means of the LPF, the effects of such parasitic resistances would be inherently compensated for.

## 3. Materials and Method

Prototypes of the circuits shown in Figure 1a,b were designed using off-the-shelf components. The OpAmp was the TLC2274 (Texas Instruments, Dallas, TX, USA), with a supply voltage of ±5 V coming from a bench-top power source (Agilent E3631A, Santa Clara, CA, USA). The bipolar input signal was provided by a waveform generator (Agilent 33210A) with a *V*_ref_ = ±1 V, *T* = 500 µs, a duty cycle of 50%, and *R*_out_ = 50 Ω. The reference resistor was equal to 1 kΩ. Consequently, the generated current was ±952 µA, which ensured low self-heating effects. The sensor resistance was emulated by resistors between 63 and 267 Ω, corresponding to temperatures between −92 and +458 °C for a Pt100 [20]. All the previous resistors were of metal film technology to have good stability with respect to temperature and time. The LPF was a passive first-order filter with a cut-off frequency of 0.16 Hz, which was implemented with a resistor of 1 MΩ and a capacitor of 1 µF.

For the diode-based circuit in Figure 1a, the prototype was implemented using a general-purpose switching diode (1N4148 from OnSemi, Phoenix, AZ, USA) for both *D*_1_ and *D*_2_. After a preliminary selection of diodes coming from the same batch, two diodes were experimentally tested at room temperature to determine the forward voltage at a forward current of 1 mA, thus resulting in *V*_F1_ = 609.9 mV and *V*_F2_ = 609.7 mV. A source and measurement unit (Agilent B2901) was employed to carry out such a measurement.

For the switch-based circuit in Figure 1b, the prototype was built using the ADG1419 (Analog Devices, Wilmington, MA, USA) as a switch, which was supplied at ±5 V. At this supply voltage, this switch has a typical on-resistance of 4.5 Ω and a mismatch between channels of 0.1 Ω, which are appropriate values for the application of interest. In addition, the selected switch offers rail-to-rail operation. The synchronization output signal of the waveform generator was connected to the logic control input of the switch to correctly control its position.

In order to experimentally evaluate the effects of the parasitic resistances of the three interconnecting wires on the circuit output, these resistances were emulated by resistors. In that sense, four scenarios were considered and tested:

(a) Scenario #1, with *R*_w1_ = *R*_w2_ = *R*_w3_ = 0. This is the reference case corresponding to no interconnecting cable between the sensor and the circuit.
(b) Scenario #2, with *R*_w1_ = *R*_w2_ = *R*_w3_ = 3.9 Ω (nominal value). This case corresponds to an interconnecting cable with a length of around 10 m, assuming a parasitic resistance of around 0.4 Ω/m [8]. The actual values of *R*_w1_ and *R*_w2_ were very similar, with a difference of around ±1 mΩ.
(c) Scenario #3, with *R*_w1_ = *R*_w2_ = 3.9 Ω, and *R*_w3_ = 2 Ω. This case emulates a strong mismatch in the resistance of wire #3.
(d) Scenario #4, with *R*_w1_ = *R*_w3_ = 3.9 Ω, and *R*_w2_ = 2 Ω. This case emulates a strong mismatch between the resistances of wires #1 and #2.

The actual value of both the resistors and the LPF output voltage (*v*_o2_) was measured by a 7 1/2-digit digital multimeter (Keysight 34470A, Santa Rosa, CA, USA). This multimeter was set in high-impedance (HZ) mode (i.e., higher than 10 GΩ) so as to avoid loading effects on the circuit output. In addition, the measurement speed of the multimeter was set with a number of power line cycles (NPLC) equal to 100, which corresponds to an integration time of 2 s, so as to have a measurement result less sensitive to potential noise and interference. On the other hand, the waveform of the voltage at the main nodes of the proposed circuits was monitored by a four-channel digital oscilloscope (Rohde&Schwarz RTB2004, Munich, Germany). An OpAmp, acting as a voltage follower, was placed at the LFP output to avoid the loading effects of the digital oscilloscope while monitoring *v*_o2_.

## 4. Experimental Results and Discussion

### 4.1. Experimental Waveforms

**Figure 2:**
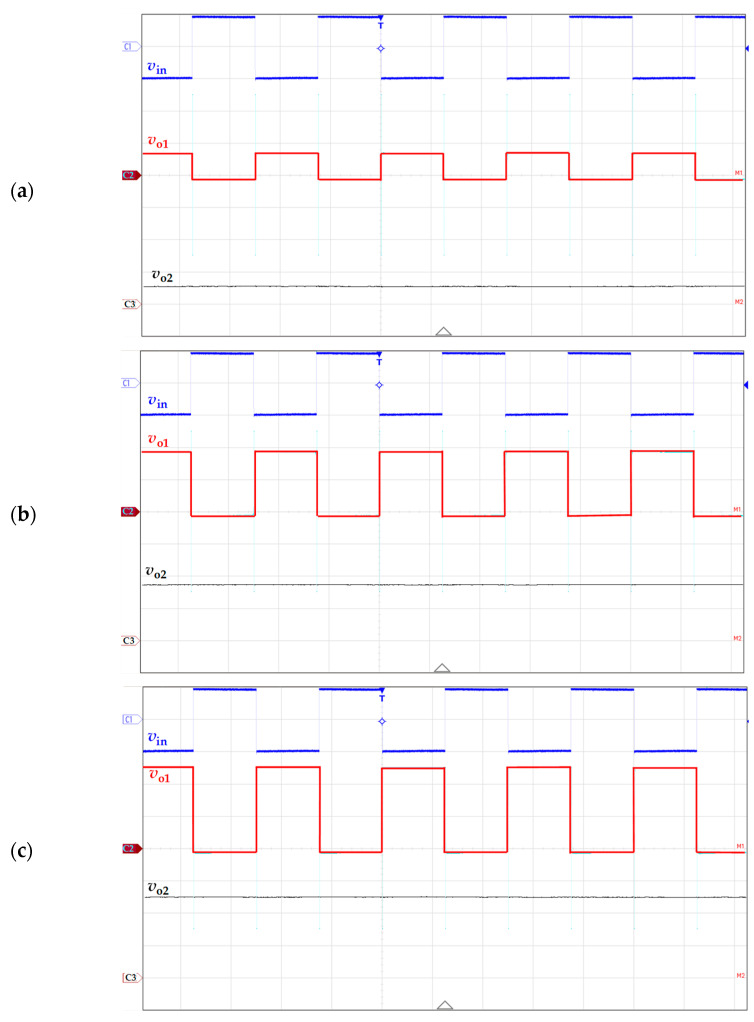
Experimental waveform of the voltage at the main nodes of the circuit proposed in Figure 1b for a sensor resistance equal to (**a**) 63 Ω, (**b**) 186 Ω, and (**c**) 267 Ω. The horizontal scale is 200 µs/div, whereas the vertical scale is 1 V/div, 100 mV/div, and 50 mV/div for *v*_in_, *v*_o1_, and *v*_o2_, respectively.

The voltages *v*_in_, *v*_o1_, and *v*_o2_ of the prototypes of the circuits in Figure 1a,b were initially monitored to check their correct operating principle. For instance, Figure 2a–c shows the waveforms of these voltages for the switch-based circuit (Figure 1b) when the sensor resistance was equal to 63, 186, and 267 Ω, respectively; for the three cases, the wire resistances were set to the conditions related to scenario #2. The experimental waveforms in Figure 2 agree with those expected theoretically and represented before in Figure 1c. An in-depth analysis of the waveforms in Figure 2 shows us the following remarks for each of the voltages monitored:

(a) Voltage *v*_in_ (with a vertical scale of 1 V/div in channel 1, and a horizontal scale of 200 µs/div): This voltage was the same for the three cases represented in Figure 2, as expected, with an amplitude of ±1 V and a period of 500 µs.
(b) Voltage *v*_o1_ (with a vertical scale of 100 mV/div in channel 2, and a horizontal scale of 200 µs/div): The amplitude of that voltage in the positive semicycle increased with increasing the sensor resistance, but that in the negative semicycle remained quite constant. This was already predicted by (7) and (6), respectively. The bandwidth of that signal was limited to 100 kHz internally in the oscilloscope to reduce the noise.
(c) Voltage *v*_o2_ (with a vertical scale of 50 mV/div in channel 3, and a horizontal scale of 200 µs/div): This voltage was a DC signal whose value increased with the sensor resistance, as predicted before by (4). It was around 30, 88, and 127 mV in Figure 2a, Figure 2b, and Figure 2c, respectively. The bandwidth of that signal was also limited to 100 kHz.

### 4.2. Diode-Based Circuit

**Figure 3:**
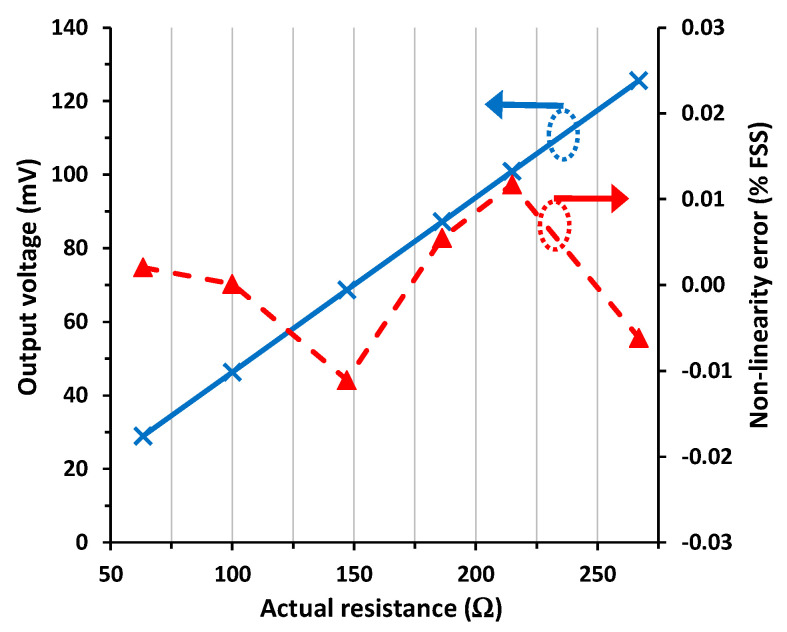
I/O characteristic of the circuit in Figure 1a for scenario #1 and the non-linearity error.

Figure 3 shows the experimental input–output (I/O) characteristic of the diode-based circuit in Figure 1a for scenario #1, which is the reference case with null parasitic resistances. The experimental output voltage in Figure 3 increased linearly with increasing the sensor resistance, as predicted before by (4). The corresponding NLE is also represented in Figure 3, which was calculated by fitting a straight line to the experimental data using the least-squares method and then expressed as a percentage of the FSS. Note that the circuit showed very good linearity with a maximum NLE of 0.012% FSS.

**Figure 4:**
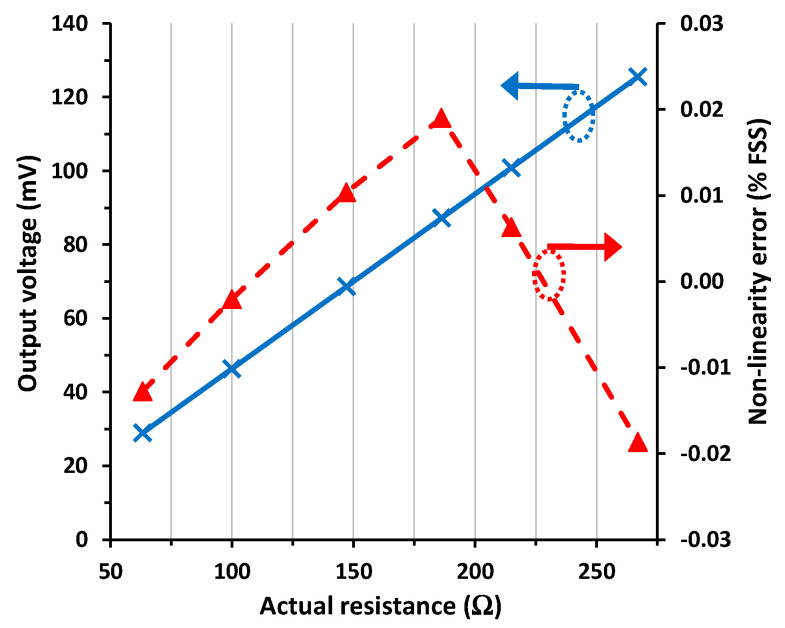
I/O characteristic of the circuit in Figure 1a for scenario #2 and the non-linearity error.

As for scenario #2 corresponding to three wires with an approximated length of 10 m, the performance of the circuit (represented in Figure 4) was very similar to that obtained before in scenario #1. The output voltage showed a maximum relative error with respect to scenario #1 of 0.02% FSS, whereas the maximum NLE was 0.019% FSS.

As for scenario #3, emulating a strong mismatch in the value of *R*_w3_ (to be precise, it was half of the value of *R*_w1_ and *R*_w2_), the resulting I/O characteristic was quite insensitive to that. Actually, the maximum relative error in the output voltage with respect to scenario #1 was 0.04% FSS, whereas the maximum NLE was 0.016% FSS.

**Figure 5:**
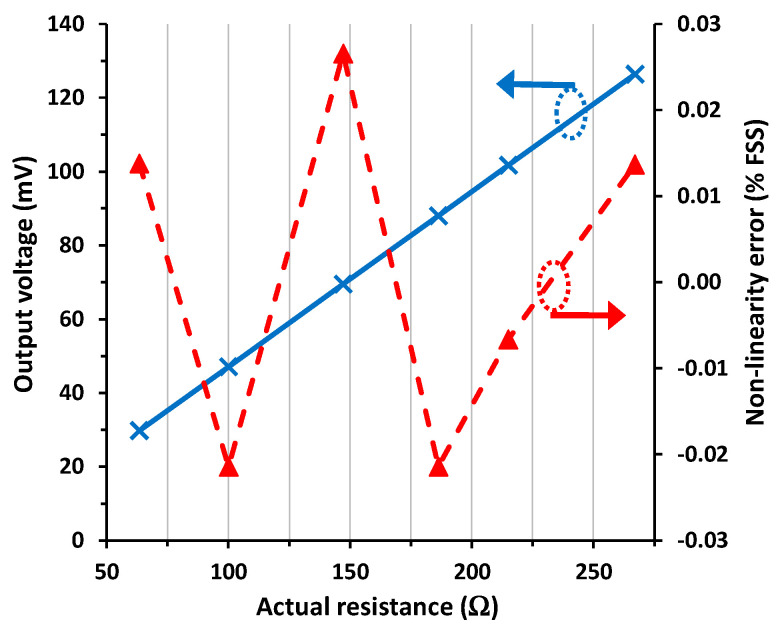
I/O characteristic of the circuit in Figure 1a for scenario #4 and the non-linearity error.

The experimental I/O characteristic for scenario #4, emulating a mismatch between *R*_w1_ and *R*_w2_, is represented in Figure 5. The output voltage was 0.85% FSS (i.e., around 0.8 mV) higher than that reported in Figure 3, which agrees with that obtained from (9) assuming that the actual value of (*R*_w1_ − *R*_w2_) ≈ 1.85 Ω. Note that the error here was quite significant since the intended mismatch was very high; to be precise, *R*_w1_ was almost twice the value of *R*_w2_. In such conditions, the NLE was slightly higher than that obtained in scenario #1, with a maximum NLE of 0.026% FSS.

### 4.3. Switch-Based Circuit

**Figure 6:**
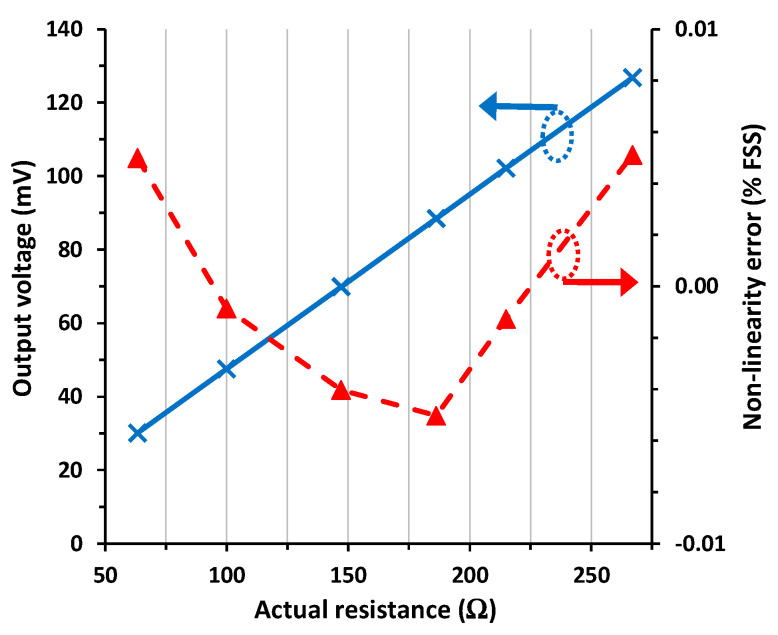
I/O characteristic of the circuit in Figure 1b for scenario #1 and the non-linearity error.

The experimental I/O characteristic of the switch-based circuit in Figure 1b for scenario #1 is represented in Figure 6. Similar to Figure 3, the output voltage in Figure 6 also increases with increasing the sensor resistance, as foreseen before by (4). In addition, the response had excellent linearity, with a maximum NLE of 0.0051% FSS (or 51 ppm), which is more than two times lower than that obtained before in Section 4.2 with the diode-based circuit.

**Figure 7:**
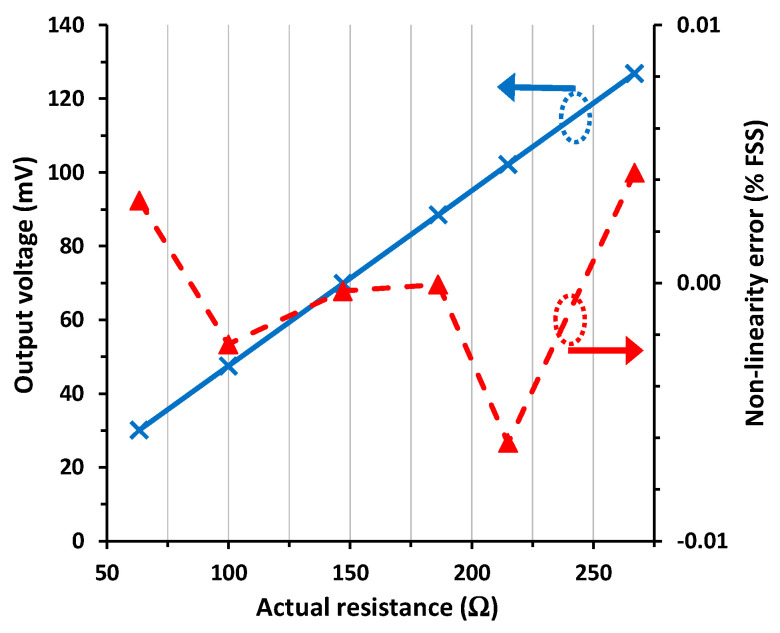
I/O characteristic of the circuit in Figure 1b for scenario #2 and the non-linearity error.

In Figure 7, the I/O characteristic of the circuit in Figure 1b under scenario #2 conditions is represented. The output voltage was almost identical to that obtained in Figure 6 with a maximum relative error (with respect to scenario #1) of 0.01% FSS, which is two times lower than that obtained in Section 4.2 under the same conditions. On the other hand, the maximum NLE was 0.0062% FSS (or 62 ppm), which is three times smaller than that found in Section 4.2.

When the circuit in Figure 1b was subjected to scenario #3, its output was practically insensitive to the mismatch affecting *R*_w3_. The maximum relative error in the output voltage with respect to scenario #1 was lower than 0.01% FSS, and the maximum NLE was 0.0071% FSS (or 71 ppm). Such values are, respectively, a factor of 4 and 2 times smaller than those obtained under the same operating conditions in Section 4.2 with the diode-based circuit.

**Figure 8:**
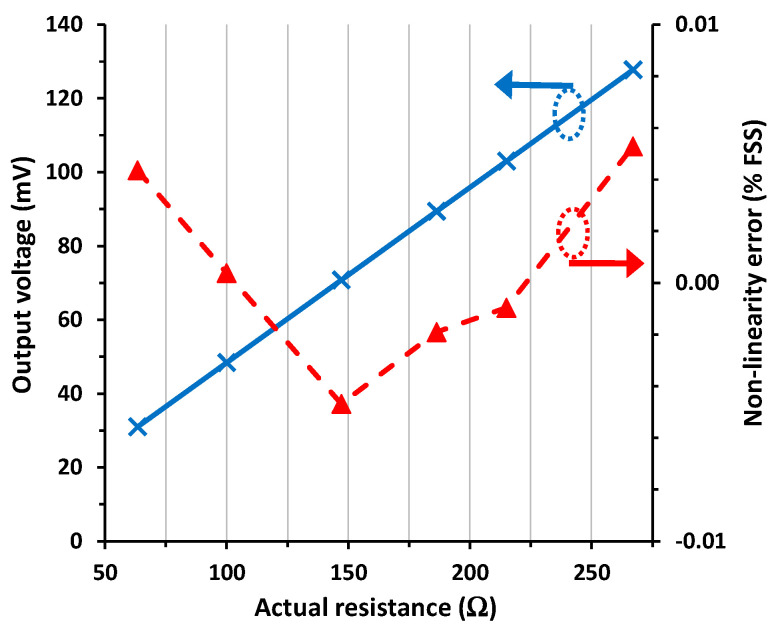
I/O characteristic of the circuit in Figure 1b for scenario #4 and the non-linearity error.

Figure 8 shows the experimental I/O characteristic for the switch-based circuit in scenario #4. In such conditions, the output voltage was around 0.9 mV higher than that obtained in scenario #1. Such an offset error corresponds to 0.9% FSS. The same as in Section 4.2, this error was mainly due to the mismatch between *R*_w1_ and *R*_w2_. Despite the presence of such an offset error, the linearity in Figure 8 remained very good, with a maximum NLE of 0.0053% FSS (or 53 ppm), which is almost the same obtained in scenario #1 (Figure 6).

The tendency of the NLE seems to correspond to a quadratic relationship between the output voltage and the actual resistance in Figure 6 and Figure 8, but this is not so clear in Figure 7. The models developed in Section 2 do not predict such a quadratic relationship, but the resulting linear models are accurate enough considering the experimental results. Note that a fitting of a quadratic relation to the experimental data in Figure 6 and Figure 8 provides a very low value of the quadratic coefficient (to be precise, 9·10^−7^). This explains the excellent linearity results obtained in these figures, with a maximum NLE of around 50 ppm.

### 4.4. Discussion

According to the experimental results reported before, the two proposed circuits offer a very remarkable linearity in spite of their simplicity, as summarized in Table 1. The maximum NLE is in the range between 0.01 and 0.03% FSS for the diode-based circuit (Figure 1a), and lower than 0.01% FSS for the switch-based circuit (Figure 1b). In addition, such a non-linearity is quite independent of the presence and also of the mismatch between the wire resistances for both circuit proposals. In comparison with the state of the art, the non-linearity of the diode-based circuit proposed herein for three-wire resistive sensors is very similar to that provided by the counterpart intended for two-wire resistive sensors [11]. Using as a reference the NLE of 50 ppm achieved here using the switch-based circuit in Figure 1b, there is an improvement of 6, 18, and 46 times with respect to the circuits suggested in [15], [12,13], and [14], respectively.

The main limitation of the two proposed circuits is that their output voltage depends on the mismatch between the parasitic resistance of wires #1 and #2, thus generating an offset error. For example, a difference between these two resistances of 1.85 Ω (which corresponds to a strong mismatch with *R*_w1_ ≈ 2·*R*_w2_) generates an offset error of around 0.8–0.9% FSS. However, this limitation was also present in other circuits (classical [18,19] and novel [12,14,15] topologies) proposed in the literature. On the other hand, the mismatch affecting wire #3 does not generate any effect on the output; this has been demonstrated theoretically and experimentally. Accordingly, wires #1 and #2 should be as similar as possible, whereas the features of wire #3 are irrelevant. It is true that circuits proposed for two-wire remote resistive sensors do not offer such a limitation in terms of the mismatch between wires #1 and #2. Nevertheless, this is at the expense of these circuits needing extra components (such as a couple of twin diodes [8,9,11] or a Zener diode [10]) at the sensor end that can indirectly suffer the extreme operating conditions of the harsh environment where the sensor is located.

## 5. Conclusions

Two implementations of a simple analog front-end circuit for remote resistive sensors with a three-wire connection have been proposed and experimentally tested. The two circuit proposals have the same operating principle, but the former relies on a couple of twin diodes, whereas the latter relies on a single-pole double-throw switch. According to the experimental results reported herein, the performance of the switch-based circuit is a factor of 2−4 times better in terms of linearity and error with respect to the reference case (i.e., with null parasitic wire resistance). For example, the maximum NLE can be as low as 0.005% FSS (50 ppm). In addition, such an excellent linearity is almost insensitive to both the presence and the mismatch between the wire resistances. The output voltage itself of the two circuit proposals also seems to be very robust to the presence of wire resistances provided that the resistances related to wires #1 and #2 are very similar, thus resulting in a relative error (with respect to the reference case) lower than 0.01% FSS in the switch-based circuit. The mismatch in the resistance related to wire #3 does not affect the measurement result. The application of such ideas to remote resistive sensors with a four-wire connection will be evaluated in the near future.

## Figures and Tables

**Table 1:** Main features of read-out circuits for the measurement of three-wire connected resistive sensors.

Ref.	Circuit Topology	Range (Ω)	Max. NLE	Mismatch Affecting the Output
[14]	Two OpAmps, two matched resistors	[80, 200]	0.24% ^1^	A single couple of wire resistances
[12]	Comparator, OpAmp, two switches, PGA	[1, 1000] k	0.09% ^2^	A single couple of wire resistances
[13]	Comparator, four switches	[80, 150]	0.09% ^3^	Two couples of wire resistances
[15]	MCU-based circuit	[60, 264]	0.03% ^1^	A single couple of wire resistances
This work	Diode-based circuit	[63, 267]	0.012% ^1^	A single couple of wire resistances
This work	Switch-based circuit	[63, 267]	0.005% ^1^	A single couple of wire resistances

^1^ Assuming a wire parasitic resistance of 0 Ω. ^2^ Assuming a wire parasitic resistance of 100 Ω. ^3^ Assuming a wire parasitic resistance of 11 Ω.

## Data Availability

The data that support the findings of this study are available upon reasonable request from the author.
